# Kasabach-Merritt syndrome: a rare cause of swelling thigh in a Cambodian newborn

**DOI:** 10.1093/omcr/omac004

**Published:** 2022-02-19

**Authors:** Chariya Chap, Sakviseth Bin

**Affiliations:** Neonatal Ward, National Pediatric Hospital, Phnom Penh, Cambodia; Neonatal Intensive Care Unit, Calmette Hospital, Phnom Penh, Cambodia

**Figure 1:**
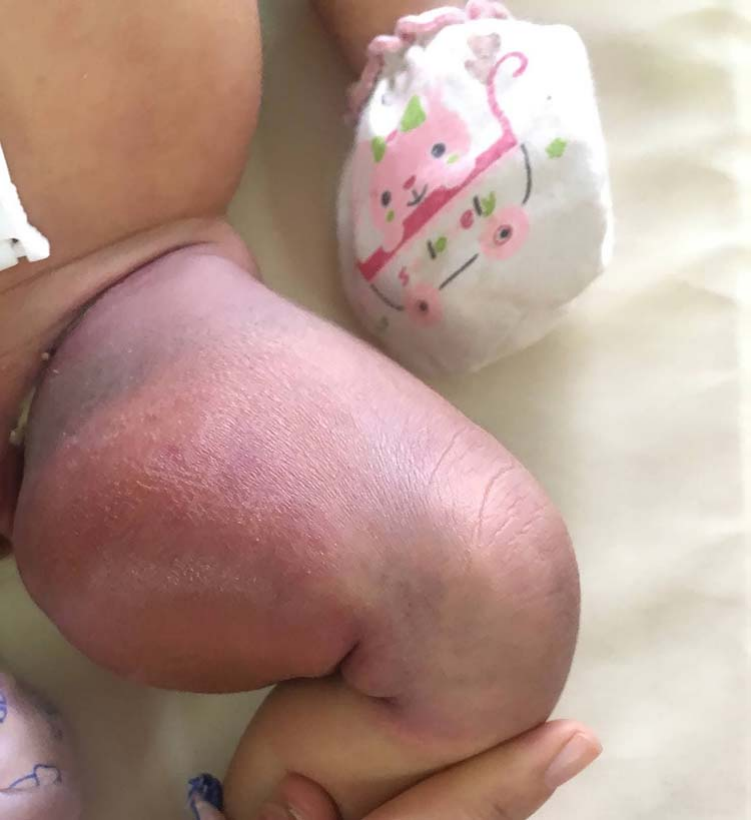
Swollen left thigh.

A 1-day-old, term infant was brought to our neonatal ward for suspected left femoral fracture. She was born vaginally at a provincial health center, with birth weight of 3000 g, without dystocia or perinatal asphyxia. The delivery was imminent with cephalic presentation. She had not been vaccinated yet. On examination, she had petechiae on the trunk and the swollen, violaceous and renitent left thigh (Fig. 1). The leg movement was reduced without a cracking sign. Babygram demonstrated neither fracture nor other defects. Ultrasound (US) of the mass suggested a voluminous hemangioma measuring 52 × 140 mm. Head, heart and whole abdomen US showed no lesions. The initial investigation revealed hemolytic anemia (hemoglobin 12 g/dl), severe thrombocytopenia (16x10^9^/l), prolonged coagulation time and reduced fibrinogen. C-reactive protein was negative, and hemoculture was sterile.

This is a rare but typical presentation of Kasabach-Merritt Syndrome (KMS), which is, to our knowledge, the first known case in our country. The vascular tumor was more likely a Kaposiform hemangioendothelioma (KHE). Tumor biopsy could not be done, and there was no indication for surgery. The patient got one transfusion of platelets and was transferred to hemato-oncology department to be treated with steroids and propranolol under close observation.

KMS was first described in 1940 in an infant with a fast-growing capillary hemangioma, severe thrombocytopenia and hypofibrinogenemia [1]. Later on, KMS was proven to be the complication of two vascular tumors: KHE (70%) and tufted angioma (10%). KHE involves mostly the extremities, with a rapidly growing tendency [2]. The median age of onset is 5 weeks [3]. The diagnosis relies on the combination of an enlarging vascular mass, consumptive thrombocytopenia and coagulopathy. Biopsy is commonly not indicated due to bleeding risks [4]. The mortality rate is about 24% [5]. The first-line treatment is pharmacological, including systemic steroids, vincristine, interferon alfa, antiplatelet agents, propranolol and sirolimus [6].
